# Tailored Dynamic Viscoelasticity of Polyurethanes Based on Different Diols

**DOI:** 10.3390/polym15122623

**Published:** 2023-06-09

**Authors:** Jiadong Wang, Min Wang, Chenxin Xu, Yang Han, Xuan Qin, Liqun Zhang

**Affiliations:** 1 State Key Laboratory of Organic-Inorganic Composites, Beijing University of Chemical Technology, Beijing 100029, China; 2 Institute of Emergent Elastomers, School of Materials Science and Engineering, South China University of Technology, Guangzhou 510640, China

**Keywords:** micro-phase separation, polyurethane, dynamic viscoelasticity, diols

## Abstract

The development of damping and tire materials has led to a growing need to customize the dynamic viscoelasticity of polymers. In the case of polyurethane (PU), which possesses a designable molecular structure, the desired dynamic viscoelasticity can be achieved by carefully selecting flexible soft segments and employing chain extenders with diverse chemical structures. This process involves fine-tuning the molecular structure and optimizing the degree of micro-phase separation. It is worth noting that the temperature at which the loss peak occurs increases as the soft segment structure becomes more rigid. By incorporating soft segments with varying degrees of flexibility, the loss peak temperature can be adjusted within a broad range, from −50 °C to 14 °C. Furthermore, when the molecular structure of the chain extender becomes more regular, it enhances interaction between the soft and hard segments, leading to a higher degree of micro-phase separation. This phenomenon is evident from the increased percentage of hydrogen-bonding carbonyl, a lower loss peak temperature, and a higher modulus. By modifying the molecular weight of the chain extender, we can achieve precise control over the loss peak temperature, allowing us to regulate it within the range of −1 °C and 13 °C. To summarize, our research presents a novel approach for tailoring the dynamic viscoelasticity of PU materials and thus offers a new avenue for further exploration in this field.

## 1. Introduction

In modern industry, there is a rising demand for elastomers that possess customized dynamic viscoelasticity to meet various requirements [1,2]. One specific application is the use of elastomers as damping materials, where the friction between molecular chains enables the conversion of kinetic energy from vibrations into thermal energy, resulting in dissipation [3,4]. The transition from a glassy state to a rubbery state in polymers is characterized by a decrease in the storage modulus and a slight increase in the loss modulus, which occurs upon reaching the material’s glass transition temperature (*T*_g_) [5]. The increase in the loss factor, defined as the ratio of the loss modulus to the storage modulus, corresponds to an enhanced damping performance of the material. The *T*_g_ of a material significantly influences its effective temperature range for damping properties [6,7]. The dynamic viscoelasticity of elastomers also plays a crucial role in the tire industry. The loss factor at 0 °C determines the wet-skid resistance, where a higher loss factor at 0 °C indicates better wet-skid resistance [8,9]. Conversely, the loss factor at 60 °C affects the rolling resistance, with a lower loss factor at 60 °C leading to reduced rolling resistance [10,11].

PU offers a wide range of adjustable *T*_g_ due to its highly designable molecular structure and the availability of various soft segments [12]. The performance of PU can be controlled by manipulating its molecular structure, which entails the utilization of Polyols [13,14], diisocyanates [15,16], chain extenders [17,18], and crosslinking agents. The soft segment, typically comprised of Polyols, including polyesters [19] and polyethers [20], determines the properties of PU, while the hard segment, consisting of isocyanates, chain extenders, and crosslinking agents, influences its mechanical strength through intermolecular interactions [21]. The dynamic viscoelasticity of PU is influenced by both the specific type of soft segment and the degree of micro-phase separation [22]. Due to the polarity difference between the soft and hard segments, they tend to self-assemble into separate micro-phase structures [23]. When there is a significant polarity difference, the interaction between hard segments becomes strong enough to generate independent hard segment domains and a higher degree of micro-phase separation. In such cases, the hard segment exerts a pronounced restrictive effect on the soft segment, thus reducing intermolecular chain friction and resulting in a lower loss factor [24]. Therefore, designing distinct molecular structures for PU and modifying the micro-phase separation structures can effectively tailor the dynamic viscoelasticity [25,26]. In the PU industry, 1,4-butanediol (BDO) is a commonly used chain extender due to its regular structure and moderate length, which facilitate the formation of PU with a regular hard segment structure [27]. However, smaller molecular glycols, such as 1,5-pentanediol (PDO), 1,6-hexanediol (HDO) [28], and diethylene glycol (DEG) [29], have not been extensively studied as chain extenders because their PUs typically exhibit inferior mechanical properties compared to BDO-based PUs.

Thermal analysis techniques, such as differential scanning calorimetry (DSC) and dynamic mechanical analysis (DMA), are effective tools for investigating the micro-phase structure of block copolymers by examining their *T*_g_ [30]. Specifically speaking, DSC measures *T*_g_ by measuring changes in the material’s heat capacity, which increases when the sample undergoes a glass transition process from a plastic to the rubbery state. DMA on the other hand, evaluates *T*_g_ by analyzing the mobility of the molecular chains. During the glass transition process, the storage modulus decreases while the loss modulus increases [31], a tendency mirrored by an increase in the loss factor. Additionally, the temperature at which the peak loss occurs in DMA reflects the degree of micro-phase separation, with a higher peak temperature indicating a lower degree of micro-phase separation. Variations in both molecular chain flexibility and the degree of micro-phase separation of PU result in different loss moduli within molecular chains. The interplay between the loss modulus and storage modulus provides an accurate representation of the interaction between different segments and the degree of micro-phase separation [32,33]. DMA yields more precise results in analyzing the degree of micro-phase separation than DSC as it combines data related to the modulus properties [34,35].

Casting polyurethane (CPU) offers the advantage of a simple processing method and finds extensive applications in damping and tire materials [36]. In this study, we synthesized a range of CPUs by exploiting poly-diols with varying flexibility as soft segments and incorporating 1,4-phenylene diisocyanate (PPDI), BDO, and trimethylolpropane as hard segments. Our investigation focused on the impacts of different soft segments on micro-phase separation structure and dynamic viscoelasticity of the CPUs. Additionally, we engineered another set of CPUs by incorporating chain extenders with different molecular weights, using polypropyl carbonate diol 221 (PCD221) as the soft segment. Through this approach, we examined the influence of various chain extenders on micro-phase separation and dynamic viscoelasticity. The research findings present an innovative methodology for tailoring the design of CPUs and achieving precise control over their dynamic viscoelasticity.

## 2. Materials and Methods

### 2.1. Materials

**Figure 1:**
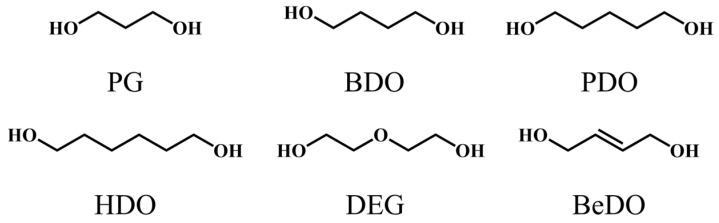
The chemical structures of the chain extenders.

Polycaprolactone diol (PCL; *M*_n_ = 2000 g/mol) was purchased from Hunan Juren Chemical Hitechnology Co., Ltd. (Yueyang, China). Polypropylene glycol adipate (PPA), Polyoxytertramethylene (PTMG; *M*_n_ = 2000 g/mol), PCD221 (*M*_n_ = 2000 g/mol), and PCD222 (*M*_n_ = 2000 g/mol) were procured from Jining Benoke Biotechnology Co., Ltd. (Jining, China). PPDI and propylene glycol (PG) were procured from Shanghai Macklin Biochemical Technology Co., Ltd. (Shanghai, China). BDO, PDO, HDO, DEG and 2-Butene-1,4-diol (BeDO) were purchased from Shanghai Aladdin Biochemical Technology Co., Ltd. (Shanghai, China). The chemical structures of the chain extenders are shown in Figure 1.

### 2.2. Synthesis Methods

Prior to synthesis, the Polyols underwent dehydration under a vacuum at 110 °C for one hour. The process started with the reaction of the Polyols and PPDI at 90 °C for one hour, resulting in the formation of a pre-polymer. The chain extenders and trimethylolpropane were then added as crosslinking agents to the pre-polymer, followed by mechanical stirring at a speed of 1000 rpm for one minute. The ratio of –NCO to –OH groups in the reaction was maintained at 1. The resulting pre-polymer was deaerated, poured into a stainless-steel mold, and cured at 100 °C for 24 h. Figure S1 provides a visual representation of the two-step synthesis process for CPUs with different soft segments and chain extenders. Finally, the CPUs were kept at room temperature for seven days before conducting the tests.

The hard segment content of the CPUs was 19%. The abbreviations used for the soft segment indicate CPUs with different soft segments. For instance, PPA-CPU represented a CPU with PPA as the soft segment. Similarly, the PCD221-CPU samples with different chain extenders were denoted by abbreviations representing specific chain extenders used. For instance, PCD221-BDO referred to a CPU where BDO was the chain extender.

### 2.3. Characterizations

A Thermo Scientific Nicolet iS10 FTIR spectrophotometer (Waltham, MA, USA) was utilized to record Fourier transform infrared spectroscopy (FTIR) spectra. The spectra were obtained using an attenuated total reflection (ATR) method with a diamond ATR accessory within the range from 4000 to 500 cm^−1^. The scan number was 32 and the resolution was 0.482 cm^−1^.

DSC measurements were performed on a DSC1 (Mettler-Toledo International, Inc., Columbus, OH, USA) scanning from −100 °C to 100 °C at a scan rate of 10 °C/min under a nitrogen atmosphere. The sample amount used ranged from 5.5 to 6.5 mg.

Atomic force microscopy (AFM) analysis was carried out using a Bruker MultiMode8 AFM (Karlsruhe, Germany) with Nanoscope IV Controller. Prior to testing, the samples were freeze-polished using an EM UC7 ultramicrotome to achieve a smooth surface. The microscope was operated at ambient temperature in PF-QNM mode, with a cantilever spring constant of 40 N/m.

Small-angle X-ray scattering (SAXS) measurements were conducted on an Xenocs Xuess2-0 system (Grenoble, France) with a λ of 0.154 nm. The sample-to-detector distance was set at 1682 mm.

The mechanical properties of the samples were evaluated under ambient conditions using a SANS instrument CMT4104 electronic tensile tester (Shanghai, China), following ASTM D638. The tests were conducted at a constant rate of 500 mm/min. The samples had a dumbbell-shaped geometry with a length of 25 mm, a width of 6 mm, and a thickness of 2 mm. Each sample was tested at least three times.

DMA (Mettler-Toledo International, Inc., Columbus, OH, USA) was performed to assess the dynamic viscoelasticity of the samples at a rate of 3 °C/min over a temperature range from −80 °C to 100 °C. The samples used for testing had a rectangular geometry with a length of 10 mm, a width of 6 mm, and a thickness of 2 mm.

## 3. Results

The first part of the study aimed to investigate the *T*_g_ and dynamic viscoelasticity of CPUs with different soft segment structures, with the goal of understanding how the variation in the soft segment influenced these properties. In the second phase, we specifically chose PCD221 as the soft segment for the CPUs due to its demonstrated highest loss peak in the first part of the study. We then explored the potential effects of different chain extenders on the *T*_g_, degree of micro-phase separation and dynamic viscoelasticity of the CPUs.

### 3.1. Characterizations of CPUs with Different Soft Segments

**Figure 2:**
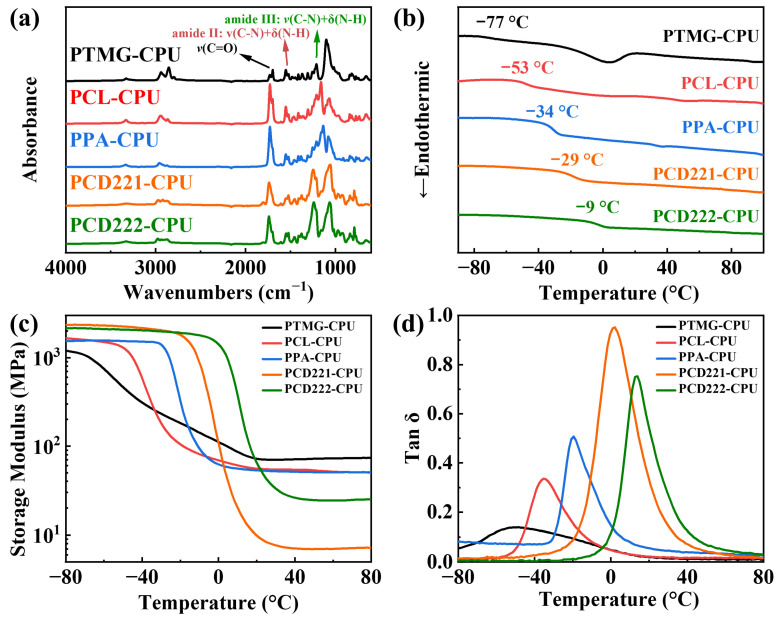
(**a**) FTIR spectra, (**b**) DSC curves, (**c**) storage modulus curves, and (**d**) tan δ curves of CPUs with different soft segments.

To regulate the dynamic viscoelasticity of CPUs across different temperature ranges, we synthesized CPUs with different soft segments. The absence of any characteristic absorption peaks of –NCO at 2260–2280 cm^−1^ in the infrared spectra of all CPUs, as shown in Figure 2a, indicates a complete reaction [37]. Moreover, we observed stretching vibration peaks at 3220–3413 cm^−1^ corresponding to the free secondary amine (N–H) and a hydrogen-bonded secondary amine. The stretching vibration peaks of ordered hydrogen-bonded carbonyl (C=O), disordered hydrogen-bonded carbonyl, and free carbonyl at 1650–1750 cm^−1^ (Figure S2) were also detected [38]. Additionally, we identified a bending vibration peak of N–H and a stretching vibration peak of C–N near 1530 cm^−1^, along with an absorption band of C–N at 1253 cm^−1^. The presence of these structures verifies the successful synthesis of CPUs. Table S1 summarizes the findings, including the utilization of Gaussian fitting and the calculation of the degree of hydrogen bonding (D_H_), which is determined by the ratio of hydrogen-bonded carbonyl groups to the total carbonyl area. The regular structures of PTMG-CPU, PPA-CPU, and PCL-CPU facilitate the formation of hydrogen bonds between hard segment molecular chains, leading to a higher D_H_ in their carbonyl groups. Notably, PTMG-CPU exhibits the highest degree of hydrogen bonding due to the absence of ester groups in the soft segment.

DSC was employed to assess the progress of glass transition and crystallization in the CPUs. The *T*_g_ of the CPUs is determined by the flexibility of the soft segments. Figure 2b illustrates the *T*_g_ values of CPUs with different soft segments, revealing the following order: PTMG-CPU < PCL-CPU < PPA-CPU < PCD221-CPU < PCD222-CPU. In theory, a higher concentration of polar groups and rigid structures in the soft segment correspond to a higher *T*_g_. It was observed that PTMG-CPU possesses the lowest *T*_g_ among all the samples, whereas PCD222-CPU exhibits the highest *T*_g_. Importantly, each individual sample retains its desirable elastomeric properties without crystallization at room temperature, indicating that the soft segments do not crystallize at the given content of the segment. The molecular structure of PTMG is regular, resulting in the melting peak of the crystalline soft segment at approximately 0 °C. Since the crystalline temperature is lower than room temperature, it does not affect the elasticity of PTMG-CPU at room temperature. The results of DMA presented in Figure 2c,d further corroborate the order of *T*_g_ values obtained through DSC. Furthermore, the DMA results validate the hypothesis that the temperature range of the loss peak and, consequently, the damping temperature range can be adjusted by selecting different soft segments.

### 3.2. Characterizations of CPUs with Different Chain Extenders

#### 3.2.1. The Degree of Hydrogen Bonding and Thermal Properties

**Figure 3:**
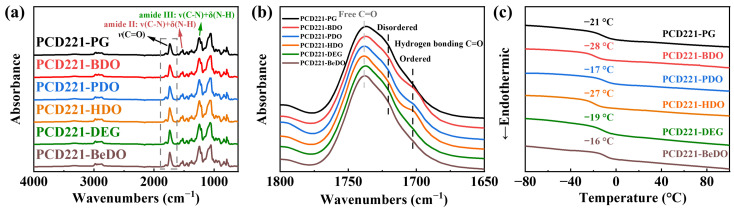
(**a**) FTIR spectra, (**b**) FTIR spectra of 1800 to 1650 cm^−1^, and (**c**) DSC curves of CPUs with different chain extenders.

In an effort to precisely modulate the dynamic viscoelasticity of the CPUs, we conducted research on a range of CPUs incorporating different chain extenders, with PCD221 utilized as the soft segment. Among the six chain extenders examined, HDO and BDO possess regular structures with an even number of carbons. PG and PDO also possess regular structures, but with an odd number of carbons. In contrast, DEG and BeDO have irregular structures. Figure 3a displays the FTIR spectra of the CPUs, each containing a distinct chain extender. Due to the relatively low proportion of chain extender within the PU, directly identifying the corresponding absorption peaks of different chain extenders is challenging. However, the influence of the various chain extenders on the formation of the hard segment is still discernible, as they lead to noticeable differences in the degree of hydrogen bonding between the soft and hard segments, as depicted in Figure 3b. The D_H_ was computed and the results are summarized in Table S2. The formation of ordered hydrogen bonds in PCD221-HDO and PCD221-BDO is significantly more extensive, while this is considerably less in PCD221-PG and PCD221-HDO. Moreover, the degree of ordered hydrogen bonds in PCD221-DEG and PCD221-BeDO is even lower. These findings suggest that chain extenders with an even number of carbon atoms in the main chain are more conducive to the formation of an ordered hard segment structure. Conversely, those with an odd number of carbon atoms tend to impede the development of the ordered hard segment structure. Additionally, irregular structures also hinder the formulation of an ordered hard segment structure.

The DSC curves of CPUs with varying chain extenders are presented in Figure 3c. The *T*_g_ values demonstrate a variation range of up to 10 °C, depending on the specific chain extender used. This fluctuation in *T*_g_ reflects the changes in the degree of micro-phase separation associated with the chain extender type. A higher degree of separation reduces the restrictive effects of the hard segment on the soft segment, resulting in a lower *T*_g_ value for the soft segment. Monomers with an odd number of carbon atoms and irregular structures tend to impede the partitioning of hard segments, leading to increased mixing between different segments and amplifying the aforementioned restrictive effect. Consequently, BDO and HDO, characterized by regular structures and an even number of carbon atoms, exhibit the lowest *T*_g_.

#### 3.2.2. Micro-Phase Separation Structure

**Figure 4:**
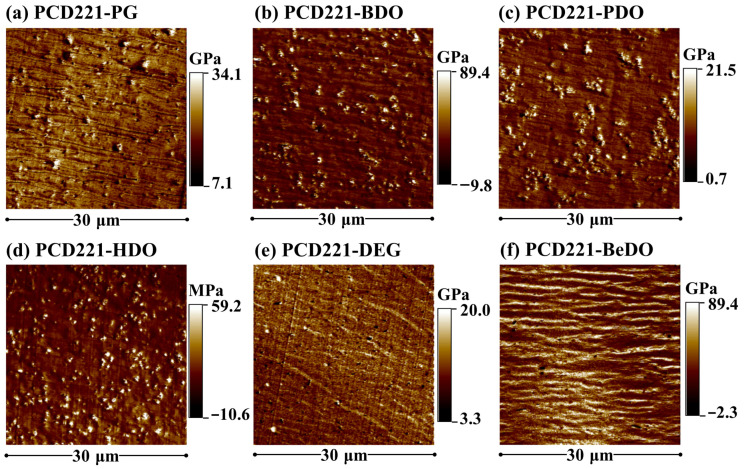
AFM modulus mapping images of CPUs with different chain extenders.

In block copolymers, the shape of the hard segment domain transitions gradually to spherical, chylindrical, bi-continuous, and lamellar structures as the content of hard segment increases [39]. Figure 4 showcases the AFM modulus mapping images of PCD221-CPUs with different chain extenders. In these images, the bright and dark regions represent the hard and soft segment domains, respectively, each characterized by high and low moduli [40]. The extent of brightness and darkness corresponds to the modulus level: the brighter the color, the higher the modulus, and vice versa. CPUs with PG, BDO, PDO, and HDO as chain extenders exhibit a spherical structure in their hard segment domains. These results clearly demonstrate the significant impact of the chain extender on the micro-phase separation structure. In the main chain consisting of repeating methylene units, the CPUs’ hard segment domain content of PG, BDO, PDO, and HDO as chain extenders notably increases with the molecular weight. As the molecular weight of the chain extender increases, the length of the hard segment increases, and the two carbamates formed by each chain extender exhibit enhanced mobility in three-dimensional space. This facilitates the formation of hydrogen bonding interactions between hard segments, leading to an enlargement of the hard segment domains through the stacking of hard segments [41]. Consequently, the content of hard segment domains observed in the AFM modulus mapping images also increases. However, in the case of PCD221-DEG and PCD221-BeDO, the hard segment domains exhibit a bi-continuous structure, indicating that irregular structures do not facilitate the development of the hard segment structure. The different micro-phase separation structures can be attributed to different phase diagrams of CPUs with different chain extenders. The emergence of these bi-continuous phases is the result of the diverse structural stacking forms of the hard segments, making it challenging to determine the degree of micro-phase separation in PCD221-DEG and PCD221-BeDO solely from AFM modulus mapping images.

**Figure 5:**
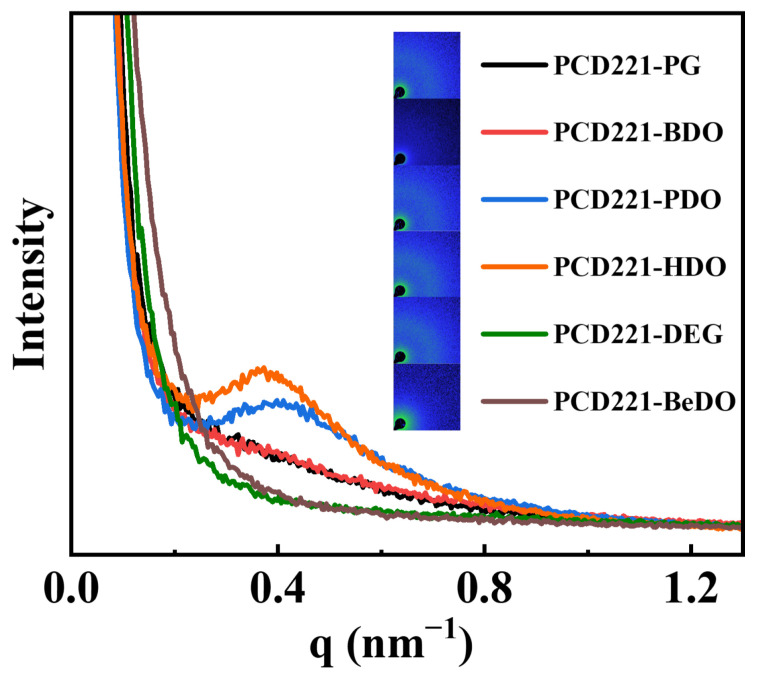
The 2D-SAXS patterns and the corresponding 1D-SAXS intensity profiles of CPUs with different chain extenders.

Figure 5 showcases the 2D and 1D SAXS spectra of PCD221-CPUs with different chain extenders. These CPUs’ micro-phase separation structures reveal the electron density difference between the ordered crystalline structure in the hard segment and the amorphous soft segment domains, giving rise to a scattering ring in the 2D SAXS curve and a scattering peak in the 1D SAXS spectrum. By applying Bragg’s law, we determined the periodicity (*D*) of the crystalline structures and summarized the results in Table S3 [42]. It was observed that CPUs incorporating structurally regular chain extenders, such as PG, BDO, PDO, and HDO, exhibit well-defined crystalline structures. CPUs with BDO and HDO as chain extenders display high scattering peaks. On the contrary, CPUs using DEG and BeDO as chain extenders with irregular structures do not exhibit noteworthy crystalline structures. These findings suggest that chain extenders with an even number of carbon atoms and a regular structure promote the formation of crystalline structures in the hard segment. As the molecular weight of CPUs incorporating PG, BDO, PDO, and HDO as chain extenders increases, the molecular chains within the hard segment lengthen, leading to an increased distance between crystalline structures and, consequently, an increased value of *D*. However, CPUs using DEG and BeDO as chain extenders do not form regular crystalline structures between hard segments, and thus the scattering ring vanishes.

#### 3.2.3. Mechanical Properties and Dynamic Viscoelasticity

**Figure 6:**
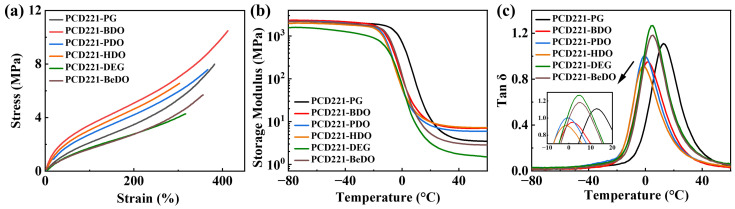
(**a**) Stress–strain curves, (**b**) storage modulus curves, and (**c**) tan δ curves of CPUs with different chain extenders.

To investigate the relationship between micro-phase separation in CPUs with different chain extenders and their macroscopic performance, mechanical properties were tested. Figure 6a illustrates the tensile strength of various PCD221-CPUs, which serves an indicator of the interaction strength between molecular chains. A comprehensive overview of the mechanical properties of CPUs with different chain extenders is provided in Table S4. When the samples were elongated by 100%, the PCD221-CPUs stress values followed the following order: PCD221-BDO > PCD221-HDO > PCD221-PDO > PCD221-PG > PCD221-DEG ≈ PCD221-BEDO. This pattern was consistently observed at 300% elongation, indicating stronger molecular chain interactions in CPUs with structurally regular diols. Particularly, PCD221-BDO exhibits the highest level of interactions and consequently the highest stress at 100% elongation. It is worth noting that due to the low content of the hard segment domain in PCD221-CPUs and the limited degree of molecular chain orientation during stretching, the overall elongation at break of the materials remains relatively low.

DMA was conducted to evaluate the dynamic viscoelasticity of PCD221-CPUs with various chain extenders. Figure 6b,c illustrates the storage modulus and loss factor curves of the CPUs, and a summary of the dynamic viscoelasticity of CPUs with each chain extenders is provided in Table S4. The material’s storage modulus directly reflects the interaction strength between the soft and hard segments. The ranking of storage modulus is as follows: PCD221-HDO ≈ PCD221-BDO > PCD221-PDO > PCD221-PG > PCD221-BeDO > PCD221-DEG. A higher storage modulus corresponds to stronger intermolecular chain interactions, which aligns with the stress at 100% elongation findings. When the interaction between the hard segments is stronger, they are more likely to separate from the soft segments, forming independent micro-regions. This leads to a higher degree of micro-phase separation and a reduced restrictive effect of the hard segment on the soft segments. Consequently, molecular chains in the soft segments exhibit higher motility, transitioning from the glassy to the rubbery state at low temperatures, reflected by a lower loss peak temperature. Therefore, the sequence of the loss peak temperatures reflects the degree of micro-phase separation: PCD221-PG > PCD221-DEG > PCD221-BeDO > PCD221-BDO > PCD221-PDO > PCD221-HDO. Among the PCD-CPUs with PG, BDO, PDO, and HDO as chain extenders, PCD221-PG demonstrates the lowest degree of micro-phase separation, while PCD221-HDO exhibits the highest. These observations align with the micro-phase separation detected in AFM modulus mapping images. The loss peak temperatures of PCD221-DEG and PCD221-BeDO fall between those of PCD221-PG and PCD221-BDO, indicating an intermediate degree of micro-phase separation. The loss factor at room temperature, which represents intermolecular chain friction, inversely correlates with the degree of micro-phase separation. PCD221-PG has the highest loss factor at room temperature due to the lowest degree of micro-phase separation and the strongest intermolecular chain friction. The results of the loss factor at room temperature further confirm the pattern of micro-phase separation observed in CPUs with different chain extenders.

## 4. Conclusions

Our study provides valuable insights into the influence of soft segments and chain extenders on CPU micro-phase separation and dynamic viscoelasticity. We have demonstrated that by adjusting the flexibility of the soft segments, the loss peak temperature of CPUs can be effectively controlled within a wide temperature range from −50 °C to 14 °C, covering a temperature range of 64 °C. For CPUs specifically synthesized with PCD221, chain extenders with a regular structure and high molecular weight tend to bolster the aggregation of hard segments, resulting in a higher degree of micro-phase separation. Notably, chain extenders with an even number of methylenes can induce self-assembly of hard segments into crystalline structures. Among the chain extenders investigated, hard segments incorporated with BDO exhibit the strongest interaction between molecular chains, resulting in CPUs with the highest modulus and stress at 100% elongation. Conversely, chain extenders with irregular structures, such as DEG and BeDO, yield CPUs with intermediate degrees of micro-phase separation between those of PCD221-BDO and PCD221-PG, primarily influenced by their molecular weights. By carefully selecting and tuning the chain extenders, we successfully modified the temperature of the loss peak in CPUs from −1 °C to 13 °C, allowing for precise control over the dynamic viscoelasticity in a temperature range of 14 °C. In summary, this research offers a novel approach for customizing the dynamic viscoelasticity of PUs and tuning the degree of micro-phase separation. This conclusion could have broad implications for synthesizing tailored polyurethane materials with specific physical properties in various fields.

## Data Availability

The data presented in this study are available on request from the corresponding author.

## Supplementary Materials

The following supporting information can be downloaded at: https://www.mdpi.com/article/10.3390/polym15122623/s1, Figure S1. Synthesize route of CPUs with different soft segments and different chain extenders; Figure S2. FTIR spectra in the range of 1650–1800 cm^−1^ of CPUs with different soft segments; Table S1. The D_H_ in the FTIR spectra of CPUs with different soft segments; Table S2. The D_H_ in the FTIR spectra of PCD221-CPUs with different chain extenders; Table S3. The periodicity (*D*) of PCD221-CPUs with different chain extenders; Table S4. Mechanical properties and dynamic viscoelasticity and of PCD221-CPUs with different chain extenders.
