# Supplementary material for: Tailored Dynamic Viscoelasticity of Polyurethanes Based on Different Diols
Source: Polymers (Basel). 2023 Jun 9;15(12):2623. doi: 10.3390/polym15122623 (PMC10302606; doi:10.3390/polym15122623)
Supplement: Supplementary file 1 [file polymers-15-02623-s001.zip › polymers-2420123-supplementary.pdf]

Supporting Information

Tailored Dynamic Viscoelasticity of Polyurethanes Based on Different Diols

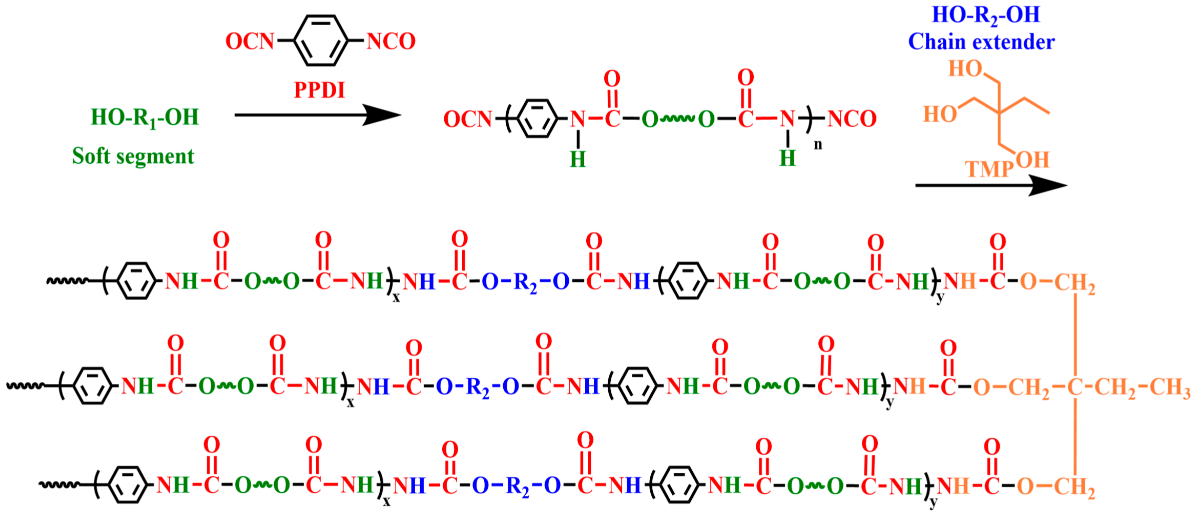

Figure S1. Synthesize route of CPUs with different soft segments and different chain extenders

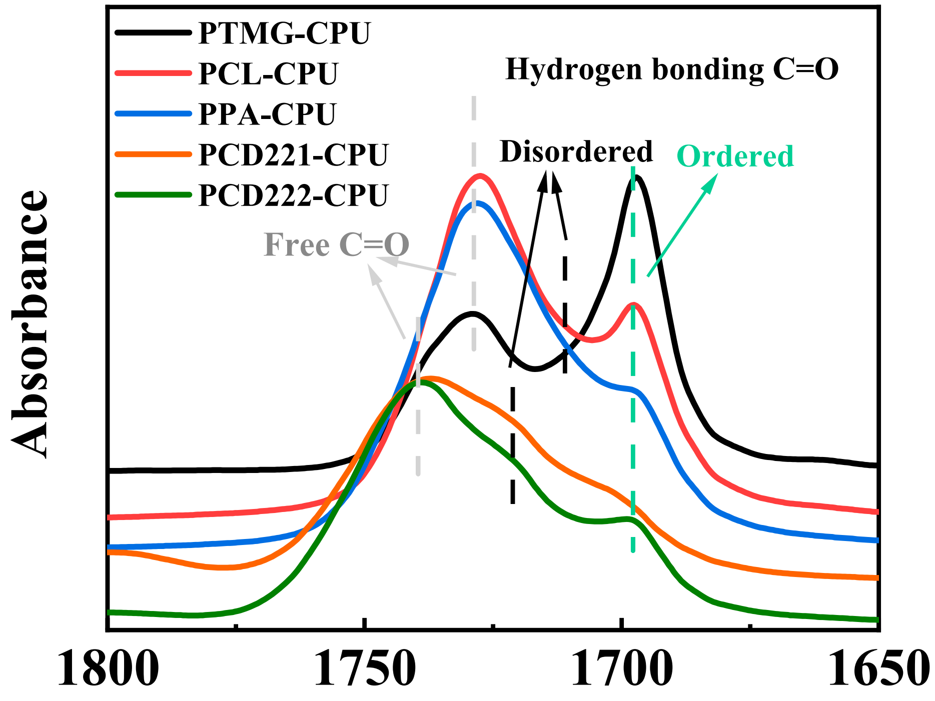

Figure S2. FTIR spectra in the range of 1650-1800 cm−1 of CPUs with different soft segments.

Table S1. The D_H in the FTIR spectra of CPUs with different soft segments.

Sample	The degree of free C=O (%)	The degree of disordered hydrogen bonding C=O (%)	The degree of ordered hydrogen bonding C=O (%)
PTMG-CPU	43.5	7.6	48.9
PCL-CPU	68.1	1.7	30.2
PPA-CPU	60.7	23.3	16.0
PCD221-CPU	59.8	2.9	37.3
PCD222-CPU	47.5	40.1	12.4

Table S2. The D_H in the FTIR spectra of PCD221-CPUs with different chain extenders.

Sample	The degree of free C=O (%)	The degree of disordered hydrogen bonding C=O (%)	The degree of ordered hydrogen bonding C=O (%)
PCD221-PG	59.5	17.3	23.2
PCD221-BDO	59.8	2.9	37.3
PCD221-PDO	65.9	8.8	25.3
PCD221-HDO	62.8	3.3	33.8
PCD221-DEG	60.3	19.9	19.8
PCD221-BeDO	64.2	16.1	19.7

Table S3. The periodicity (*D*) of PCD221-CPUs with different chain extenders.

Sample	q_{max} (nm^{-1})	*D* (nm)
PCD221-PG	0.4403	14.27
PCD221-BDO	0.4313	14.57
PCD221-PDO	0.4264	14.74
PCD221-HDO	0.3780	16.62
PCD221-DEG	-	-
PCD221-BeDO	-	-

Table S4. Mechanical properties and dynamic viscoelasticity and of PCD221-CPUs with different chain extenders.

Sample	Tensile Strength (MPa)	Elongation at Break (%)	Stress at 100% elongation (MPa)	T_g (°C)	Tan δ_{max}
PCD221-PG	8.0	382	2.13	13	1.110
PCD221-BDO	10.5	412	3.46	2	0.949
PCD221-PDO	7.6	367	2.75	-1	1.002
PCD221-HDO	6.6	303	3.07	-1	0.912
PCD221-DEG	4.3	317	1.74	5	1.268
PCD221-BeDO	5.7	357	1.65	1	1.184
